# Repigmentation and hair regrowth in perinevoid alopecia treated with topical gabapentin: A case for neuromodulation

**DOI:** 10.1016/j.jdcr.2026.04.068

**Published:** 2026-05-11

**Authors:** Marcelo Rigatti, Maiby De Bastiani, Isabel Crivelatti, Débora Cadore, Tatiana de Medeiros

**Affiliations:** Department of Dermatology, University Hospital, Federal University of Santa Catarina (HU-UFSC), Florianópolis, Brazil

**Keywords:** alopecia areata, gabapentin, neuromodulation, perinevoid alopecia, scalp pruritus

Perinevoid alopecia is a rare alopecia areata (AA)-like condition characterized by a localized, non-scarring alopecic patch surrounding a melanocytic nevus. It is thought to result from an autoimmune response against shared melanocytic and follicular antigens, placing it at the interface of follicular autoimmunity and pigmentary disturbance.^1^ Surgical excision is generally considered the treatment of choice; however, evidence supporting noninvasive therapies remains limited. We report a case of perinevoid alopecia successfully treated with topical gabapentin, with improvement in pruritus, hair density, and hair shaft pigmentation.

## Case report

**Fig 1:**
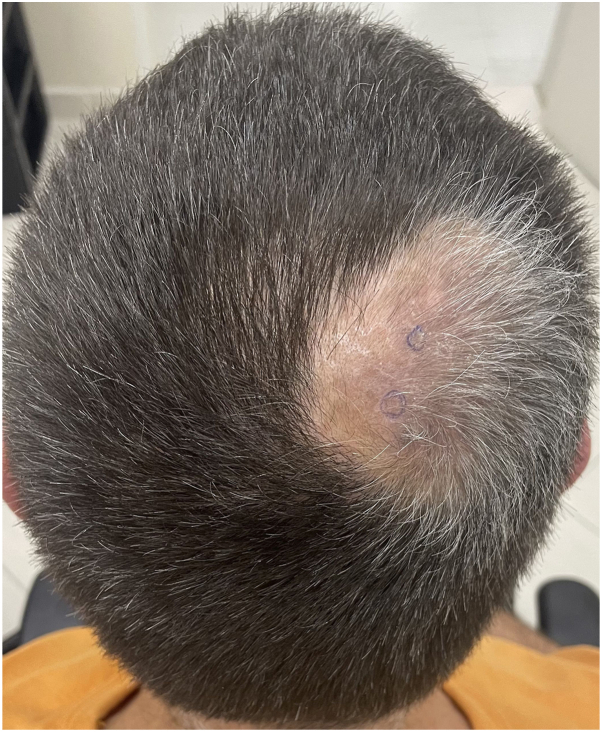
Baseline clinical image showing a localized alopecic patch with perinevoid hair rarefaction surrounding a melanocytic nevus.

A 44-year-old man presented with a 3-y history of localized alopecia in the parieto-occipital scalp, associated with white hairs surrounding a pigmented nevus (Fig 1).

The lesion had been present since birth. Medical history was unremarkable. Biopsies demonstrated a combined blue and intradermal melanocytic nevus, with dermal nests, fibrosis, follicular miniaturization, and a perifollicular lymphocytic infiltrate involving the upper follicle.

Baseline dermoscopy showed decreased hair density, with increased vellus hairs, and mild perifollicular scaling without scarring. Serologic testing for syphilis and HIV and fungal studies were negative and performed as part of the diagnostic workup to exclude infectious causes of localized alopecia.

Prior treatments included topical and oral minoxidil, clobetasol lotion, ketoconazole shampoo, and intralesional triamcinolone, with minimal improvement and persistent pruritus. Topical gabapentin 6% lotion (pH 5.2), compounded in a hydrophilic vehicle, was initiated twice daily, without concomitant therapies.

**Fig 2:**
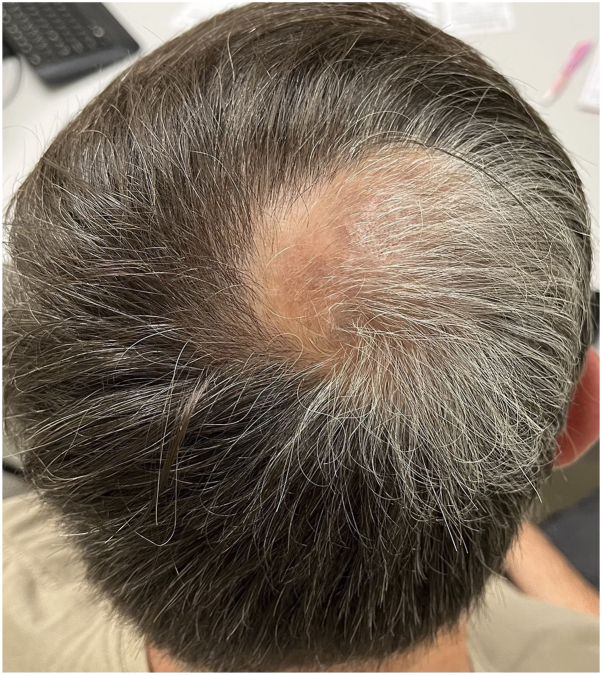
Clinical image after 6 months of topical gabapentin treatment demonstrating partial hair regrowth and reduction of the alopecic area.

After 6 months, the patient reported marked pruritus relief, with visible hair regrowth and partial repigmentation (Fig 2). Dermoscopy showed increased follicles with terminal hairs and reduced perifollicular scaling. Response was assessed clinically and dermoscopically.

## Discussion

Perinevoid alopecia is considered a localized AA variant associated with a melanocytic nevus, likely due to immune targeting of shared antigens between nevus cells and follicular melanocytes.^1^ Clinically, it presents as a well-demarcated non-scarring alopecic patch with hair depigmentation.^1^ Although excision is often curative, it may not be feasible in all cases.

Neurogenic inflammation may also contribute to inflammatory alopecias. Chronic pruritus is linked to C-fiber sensitization and neuropeptide release affecting the follicular microenvironment.^2^

Gabapentin, used for neuropathic pain and pruritus, inhibits voltage-gated calcium channels and reduces excitatory neurotransmitter release.^3^ Topical formulations may modulate cutaneous innervation and neuroimmune interactions with minimal systemic absorption.^4^

This neuromodulatory effect may contribute to improvement of the perifollicular environment, potentially supporting follicular and melanocyte function.^3^^,^^4^ Experimental data also suggest antioxidative and cytoprotective effects contributing to repigmentation and follicular resilience.^5^

This is the first report of topical gabapentin in perinevoid alopecia. Improvement in pruritus, hair density, and pigmentation supports neuromodulation as a potential therapeutic pathway when surgery is not feasible.

## Conclusion

Topical gabapentin may represent a promising noninvasive therapeutic option for perinevoid alopecia, particularly in patients with refractory pruritus. Further studies are needed to clarify its mechanisms and role in AA-related disorders.

The patient provided written informed consent for publication of clinical details and images.

### Declaration of generative AI and AI-assisted technologies in the writing process

Artificial intelligence was not used in the writing, analysis, or preparation of this manuscript.

## Conflicts of interest

None disclosed.
